# Possible Effects of Perchlorate Contamination of Drinking Water on Thyroid Health

**DOI:** 10.1155/2020/5208657

**Published:** 2020-05-13

**Authors:** Sunny P Orathel, Ronnie Thomas, N. Chandramohanakumar, Joy Job Kulavelil, Krishnapillai Girish Kumar, Vadayath Usha Menon, P. Jayaprakash, Sajitha Krishnan, P. S. Manju, Shaiju Param, M. G. Rajamanickam, U. G Unnikrishnan, Joe Thomas, Ponnu Jose

**Affiliations:** ^1^Medicine and Pulmonology, Rajagiri Hospital, Aluva, Ernakulam, Kerala, India; ^2^Department of Community Medicine, Jubilee Mission Medical College and Research Institute, Thrissur, Kerala 680005, India; ^3^Department of Oceanography, CUSAT, Kochi, Kerala, India; ^4^Kerala State Fee Regulatory Commision for Professional Colleges, Government of Kerala, Kerala, India; ^5^Department of Applied Chemistry, CUSAT, Kochi, Kerala, India; ^6^Centre for Endocrinology and Diabetes, Amrita Institute of Medical Sciences, Kochi, Kerala, India; ^7^Department of Endocrinology, Medical Trust Hospital, Kochi, Kerala, India; ^8^Department of Biochemistry, Amrita Institute of Medical Sciences, Kochi, Kerala, India; ^9^Kerala Health Services, Government of Kerala, Kerala, India; ^10^CUSAT, Kochi, Kerala, India; ^11^Indian Administrative Service, Government of Kerala, Ernakulam, Kerala, India; ^12^Biostatistics, Jubilee Mission Medical College and Research Institute, Thrissur, Kerala, India

## Abstract

**Background:**

Perchlorate is an anion that occurs as a contaminant in groundwater. It originates from the improper disposal of ammonium perchlorate, a component of rocket fuel. The objective of this study was to explore whether the exposure to perchlorate in drinking water had an impact on the thyroid function of the population residing near an ammonium perchlorate plant in Kerala. *Methodology*. Using an ecological study design, we compared the serum levels of thyroid-stimulating hormone, thyroxine, and thyroid peroxidase antibodies among a representative sample of 289 study subjects from the area surrounding the ammonium perchlorate enrichment plant to 281 study subjects in a control area.

**Results:**

The perchlorate concentration in the groundwater varied from 1600 ppb to 57,000 ppb in the 10 samples from the contaminated area and was below 24 ppb in all locations in the control area. No significant differences were found in the mean serum TSH concentration and mean T4 levels between the subjects from the contaminated area and the control area. On regression analysis, perchlorate contamination was not found to be a significant predictor of TSH.

**Conclusion:**

This study did not find any significant association between perchlorate in drinking water and changes in thyroid hormone levels. Our findings indicate the need for further investigation of this hypothesis using urinary perchlorate as a measure of individual exposure.

## 1. Background

Ammonium perchlorate is an inorganic compound widely used as an oxidizer component of propellants in solid rocket fuel. [1] It has been used therapeutically for clinical indications like hyperthyroidism associated with amiodarone toxicity [2]. In pharmacologic doses, perchlorate ions concentrate in the thyroid tissue and are competitive inhibitors of the sodium/iodine symporter (NIS). NIS is a membrane protein located on the basolateral side of the follicular cells of the thyroid gland. Perchlorate anions will decrease the active transport of iodine into the follicular cells, inhibiting organification of iodide and consequently decreasing thyroid hormone synthesis [3, 4]. This in turn increases the TSH levels causing thyroid hypertrophy or hyperplasia possibly followed by clinical hypothyroidism. [3, 4] *In vitro* studies on NIS-expressing cells by Tonacchera et al. demonstrated that perchlorate has a 30-fold greater potency for NIS than that for iodide [5].

Available evidence does not support a causal relationship between changes in thyroid hormone levels and the environmental levels of perchlorate exposure. [6] However, human activities have led to the widespread presence of perchlorate in the environment and drinking water. One major source of contamination is the manufacture and improper disposal of ammonium perchlorate that is used for rocket fuel [7]. Foetuses and infants are most vulnerable to these effects because they need thyroid hormone for normal neurodevelopment [3].

In the year 2014, a medical officer of a Primary Health Centre in Keezhmad in the Ernakulam district of Kerala noticed an unusually high incidence of hypothyroidism and subclinical hypothyroidism among the residents of the Kulakkad colony in the Keezhmad panchayath. As the Kulakkad colony shares a compound wall with the central government's Ammonium Perchlorate Experimental Plant (APEP) facility, exposure to perchlorate was suspected to be the cause of hypothyroidism. A study conducted by the National institute of Interdisciplinary Science and Technology, Thiruvananthapuram, revealed high levels of perchlorate contamination in ground and surface water around the Ammonium Perchlorate Experimental Plant (APEP) at Aluva in the Ernakulam district of Kerala, India. The contamination was found to be severe in groundwater as compared to surface water (7270 *μ*g/L vs 355 *μ*g/L) at this location. [8] Following press reports on the contamination of drinking water and the incidence of hypothyroidism, the district authorities initiated administrative action to control the situation and constituted a technical expert committee to assess the risk from these exposures. The present study aimed to examine whether exposure to perchlorate in drinking water has an impact on the thyroid function of the population residing near the ammonium perchlorate plant.

## 2. Materials and Methods

### 2.1. Study Design and Study Setting

This study used an ecologic design, integrating environmental measurement data of perchlorate levels in the groundwater and data on the thyroid function of the subjects. The study population encompasses residents from two contaminated areas that surround the Ammonium Perchlorate Experimental Plant (APEP) facility and two control areas located 20 kilometers north of the contaminated area. The areas considered as “contaminated areas” were Edathala and Keezhmad panchayaths with perchlorate levels exceeding 24 ppb in the groundwater used for drinking purpose. The areas designated as “control areas” were Pallissery and Karukutty panchayaths with perchlorate levels below 24 ppb. The authors compared the thyroid function of the subjects living in the contaminated areas to the control areas. Both areas are similar in size and other sociodemographic characteristics.

### 2.2. Sample Size, Sampling Technique, and Eligibility

We recruited a representative sample of 570 by stratified random sampling. Among them, 289 residents were living in the “contaminated areas” and 281 residents were living in the control areas. To ensure adequate representation of both genders and all age groups, the stratification was done by gender and 10-year age group. Written informed consent was obtained from each participant. As low iodine levels had potential to act as a confounder in the association of perchlorate with free T4 and TSH, participants with urinary iodine <100 *μ*g/L were excluded. Study participants who are pregnant, participants with known autoimmune thyroid disorders, and those taking medications known to affect thyroid function like levothyroxine, propylthiouracil, or beta-blockers were also excluded. After exclusion, a total of 542 subjects were found to be eligible.

### 2.3. Study Procedure and Environmental Exposure Assessment

All subjects included in this analysis received a standardized clinical examination, and a fasting blood sample was collected from which serum TSH levels, serum-free T4 levels, and TPO antibody levels were measured. The analysis of thyroid function parameters was done using the ARCHITECT i2000SR immunoassay analyser based on the chemiluminescence assay, and their normal reference ranges are as follows: serum TSH (0.35 to 4.9 uIU/ml), serum-free T4 (0.7 to 1.48 ng/dl), and TPO antibody (0.0 to 5.61 U/ml). The urinary iodine levels of the participants were also measured in the morning urine samples. As thiocyanate, which is elevated in smokers, and nitrate in drinking water have the potential to interact with iodine uptake, we ascertained the smoking status of the subjects and the nitrate levels in the groundwater samples [5].The groundwater samples were collected from eight open wells and two borewells from Edathala and Keezhmad, the contaminated areas, and from four open wells and one borewell of Pallissery and Karukutty, the control areas. The analysis for perchlorate was performed using the ion chromatography system (IC-1100, Dionex) at NIIST, Thiruvananthapuram. General physical and chemical water quality parameters including nitrate levels were analysed at the Department of Oceanography, CUSAT.

### 2.4. Statistical Analysis

For normally distributed variables, univariate analyses using the chi-square test, independent sample's *t*-test, and Pearson's correlation were done. Mann–Whitney *U* test was done for nonnormally distributed variables. The T4 values were normally distributed and the TSH values were approximately normally distributed, but the TPO antibody values were nonnormal in distribution. TSH was log transformed and analysed. Regression analyses using the generalized linear model were conducted to determine the independent associations of thyroid function with presence of perchlorate in drinking water. The following variables were included in the regression model with TSH level as the dependent variable: presence of perchlorate contamination, age, gender, and BMI (for subjects above 18 years).

## 3. Results

The total number of subjects after exclusion was 542 with 272 subjects from the contaminated area and 270 subjects from the control area. The demographic characteristics of the study population are presented in Table 1. The mean age of the study participants in the contaminated area was 34.76 ± 21.60 and 34.20 ± 19.57 in the control area. The primary source of drinking water, as participants reported, was water from the well and borewell water. The data indicate that the study subjects from the contaminated area and the control area are comparable with respect to their demographic characteristics.

We determined the perchlorate concentrations in the groundwater of ten locations from the contaminated areas and five locations from the control areas. The perchlorate concentration in the groundwater varied from 1600 ppb to 57,000 ppb in the 10 samples from the contaminated areas. These values were about 70 to 480 times higher than the permitted drinking water equivalent level of 24 ppb established by the USEPA. [7] The highest perchlorate levels were detected in the samples taken from areas adjacent to the APEP plant. The concentrations of perchlorate in the control area were largely comparable to levels in the groundwater as previously reported from other parts of Kerala [8]. The concentrations there varied from below detectable levels (BDL) to 20 ppb, all of which were less than the regulatory standard [7]. The details of the perchlorate levels in the contaminated and the control areas are summarized in Table 2.

The urinary levels of iodine in this population were also evaluated, and all the subjects included had urinary iodine >100 *µ*g/L, a level which indicated adequate iodine nutrition. [9] Nitrate and thiocyanate are naturally occurring goitrogens. The levels of nitrate found in the groundwater samples in this study were comparable to the levels found previously in samples from similar populations [7]. The proportion of smokers, which is a proxy indicator of thiocyanate exposure, among the subjects from the two areas was also similar.

The mean serum TSH concentrations were 2.049 mIU/L among the subjects from the contaminated area and 1.90 mIU/L among the subjects residing in the control area. This difference was not statistically significant (*p* value 0.657). Expressing the data as proportions, there was no significant difference in the prevalence of hypothyroidism, which was 4% in the contaminated area and 3.3% in the control area (*p* value 0.841). We repeated the analysis after excluding subjects with abnormal TPO levels. The mean serum TSH concentrations after exclusion were 1.827 mIU/L among the subjects from the contaminated area and 1.780 mIU/L among subjects residing in the control area. This difference was also not statistically significant (*p* value 0.671). The results were consistent when stratified by age, gender, and BMI. The details of the thyroid function parameters with respect to the area are given in Tables 3 and 4.

Adjusting for age, gender, and BMI (for subjects above 18 years) and modeling TSH concentration as the outcome, we observed no significant relationship with perchlorate levels in the generalized linear regression model. The *B* coefficient for perchlorate contamination was −0.022 (*p* value = 0.62). However, age of the participants was found to be a significant predictor of thyroid function. The results of the regression analysis are shown in Table 5.

## 4. Discussion

Our study did not find any evidence for the increased incidence of abnormal thyroid function among the study subjects who resided in communities with perchlorate-contaminated groundwater. The results of our univariate analysis revealed a slightly increased prevalence of hypothyroidism with exposure to perchlorate levels above 24 ppb in drinking water, but this was not statistically significant. The geometric mean of primary thyroidal parameters TSH and T4 showed no significant differences between the subjects from the perchlorate-contaminated area and the control area. These findings were consistent with the majority of available epidemiological evidence from chronic occupational exposure studies and ecologic investigations [2].

The majority of the available published scientific literature does not demonstrate a causal association between perchlorate exposure and hypothyroidism. A study by Li et al. on the prevalence of thyroid diseases in Nevada counties did not observe an increased rate of any specific thyroid disease associated with perchlorate exposure in drinking water [10]. A similar study on thyroid function in the pediatric population of a perchlorate-contaminated area showed that there was no variation in the TSH levels and free thyroxine (T4) levels as compared to children of other regions with no perchlorate in drinking water. [11] The possible explanation why long-term exposure to high levels of perchlorate did not affect plasma TSH levels or T4 levels, as suggested by De Groef et al., is that the inhibition of iodine uptake is duration dependent and the existence of compensatory mechanisms which counteract the perchlorate-induced NIS inhibition [12]. Similar observations were made in an experimental setting which observed no effect on the inhibition of iodide uptake by the thyroid at 0.007 mg/kg of perchlorate per day. [4] An observational study carried out at an ammonium perchlorate production factory in China found no effect on thyroid function from long-term exposure to ammonium perchlorate. [13].

However, it must be pointed out that some investigators have identified a relationship between perchlorate contamination of water supplies and thyroid dysfunction. The results of the present study is in contrast with the findings of a cross-sectional study based on the NHANES data by Blount et al. which conclude that perchlorate exposure is associated with increased TSH and decreased T4 among women who had iodine deficiency (urinary iodine values < 100 *μ*g/L) [14]. Similar observations were made by Greer et al. in the TSH levels of subjects whose iodine nutrition was insufficient [4]. The observations found that intake of 150 *μ*g iodine daily would protect against goiter in a person whose perchlorate ingestion is 4 mg/day (dose that would inhibit iodide uptake) [4]. However, the present study excluded individuals who had urinary iodine values < 100 *μ*g/L as this may have a confounding effect on the outcome being assessed [9]. This can be one reason why the authors of the present study did not observe any significant effect for perchlorate. The findings of another study which looked at the relation between perchlorate and serum-free thyroxine (FT4) indicate that adolescent boys and girls are vulnerable to the thyroid-blocking effects of perchlorate [15].

Pregnancy is considered to be a vulnerable period for perchlorate exposure, and it may have an adverse effect on the offsprings' cognitive development. A study conducted among Arizona newborns by Brechner et al. reported an association between perchlorate in drinking water and incidence of hypothyroidism [16]. A study by Taylor et al. in 2014 among 846 women at gestational age 16 weeks reported an association between urinary perchlorate and offspring IQ at 3 years of age. [17] The transport of iodine into breast milk is through the same sodium/iodine symporter which is inhibited by the perchlorate ion [18]. Pregnant mothers were excluded from the present study.

The results of the current study were in agreement with those of previous studies with respect to the confounding effects of demographic factors. Among the demographic characteristics we analysed in the regression model, only age was found to be a significant factor in predicting high TSH. In the healthy adult population, mean TSH increases with age [19]. Although many studies report a positive association between serum TSH within the normal range and BMI, we did not find any significant association in the regression model [20, 21].

One possible explanation for the findings of the present study is that the exposure ascertainment procedure did not account for variation due to individual consumption patterns of water. Even though all residents in the contaminated area reported consuming groundwater from the area, the possibility of them relying on several water sources cannot be ruled out. This was a major limitation of the study, and this can be rectified only by individual measurement of perchlorate exposure using urinary perchlorate estimation.

### 4.1. Recommendations

1. Biomonitoring of perchlorate exposure in this population by measuring urinary perchlorate levels.
2. Routine environmental surveillance of perchlorate levels in the groundwater.
3. Small amount of iodine supplementation can modify the goitrogenic effects of perchlorate in drinking water [22].
4. Analytical research studies which measure individual exposures such as urinary perchlorate with the additional use of indirect biomarkers for perchlorate.
5. Measures to educate the residents on proper storage of iodised salt at home so as to maintain the iodine content at 15 ppm.

## 5. Conclusion

The authors concluded that the data from the current ecological study was not consistent with a causal association between thyroid function and exposure to perchlorate in the groundwater used for drinking purpose. However, the present study is subject to ecological fallacy. We carried out the study under the assumption that the geographically delineated exposures to perchlorate validly represent those of the population in the contaminated area. Further investigation of the hypothesis is warranted using analytical studies that incorporate individual measurement of perchlorate exposure in the contaminated area using urinary perchlorate estimations.

## Figures and Tables

**Table 1:** Demographic characteristics of the study population.

Characteristic		Contaminated area *n* (%)	Control area *n* (%)
Gender distribution	Males	138 (50.7)	135 (50)
	Females	134 (49.3)	135 (50)
Age distribution (years)	0–20	79 (29)	85 (31.4)
	21–40	82 (30.1)	76 (28.1)
	41–60	70 (25.7)	84 (31.1)
	61–80	41 (15.1)	25 (9.3)

**Table 2:** Distribution of perchlorate concentration in groundwater samples from the study areas.

Area	Location (source)	Perchlorate (ppb)
Contaminated area	1. Keezhmad (open well)	9000
	2. Keezhmad (open well)	13000
	3. Kulakkad (open well)	53000
	4. Nalamile (open well)	1600
	5. Edathala (open well)	17000
	6. Keezhmad (borewell)	2000
	7. Edathala (borewell)	7800
	8. Societypady (open well)	4700
	9. APEP (open well)	57000
	10. Meekarankunnu (open well)	1800

Control area	11. CHC, Pallissery (open well)	20
	12. Pallissery (open well)	BDL
	13. Elavoor kavala (open well)	14
	14. Karukutty (open well)	BDL
	15. SCMS (borewell)	5

**Table 3:** Comparison of mean values of TSH, T4, and TPO antibodies measured among the study participants based on the study area.

Thyroid function	Contaminated area	Control area	*p* value
TSH (mean ± SD)	2.049 ± 1.19	1.907 ± 1.16	0.657
Free T4 (mean ± SD)	1.079 ± 0.138	1.084 ± 0.176	0.696
TPO antibody (median)	0.450	0.605	0.053

**Table 4:** Thyroid status of subjects from the contaminated and control areas (Univariate analysis).

Thyroid status of subjects	Contaminated area	Control area	*p* value
	*n* (%)	*n* (%)	
Hypothyroid	11 (4.1)	9 (3.3)	0.740
Euthyroid	257 (94.4)	255 (94.5)		
Hyperthyroid	4 (1.5)	6 (2.2)		

**Table 5:** Regression analyses of association between perchlorate and TSH.

Independent variable	Coefficient	SE	*p* value
Intercept	2.133	0.239	<0.001
Age	0.143	0.003	0.002
Gender	−0.004	0.104	0.939
BMI	−0.095	0.024	0.498
Perchlorate contamination	−0.022	0.102	0.629

## Data Availability

The data used to support the findings of this study can be made available on request.

## Abbreviations

NIS: Sodium/iodine symporter
TSH: Thyroid-stimulating hormone
APEP: Ammonium Perchlorate Experimental Plant
ppb: Parts per billion
T4: Thyroxine 4
TPO: Thyroid peroxidase
NIIST: National Institute for Interdisciplinary Science and Technology
CUSAT: Cochin University of Science and Technology
BMI: Body mass index
USEPA: United States Environmental Protection Agency
BDL: Below detectable levels
NHANES: National Health and Nutrition Examination Survey
IQ: Intelligence quotient
ppm: Parts per million.
